# Commentary: Does Lithium Deserve a Place in the Treatment Against COVID-19? A Preliminary Observational Study in Six Patients, Case Report

**DOI:** 10.3389/fphar.2020.613734

**Published:** 2020-12-16

**Authors:** Christopher E. Rudd

**Affiliations:** ^1^ Centre de Recherche- Maisonneuve-Rosemont Hospital (CR-HMR), Montreal, QC , Canada; ^2^ Département de Medicine, Université de Montréal, Montreal, QC, Canada; ^3^ Department of Medicine, Division of Endocrinology & Medical Biochemistry, McGill University Health Center, Montreal, QC, Canada

**Keywords:** lithium, COVID-19, GSK-3, SARS CoV2, viral replication

The manuscript by Spuch et al., provides interesting preliminary data in support of a possible role of lithium the treatment of patients with COVID-19 (Spuch et al., 2020). Although limited to six patients treated with different additional therapeutic approaches, it was encouraging that the authors found the lithium carbonate increased lymphocyte numbers, reduced plasma reactive C-Protein levels while decreasing the neutrophil-lymphocyte ratio used as a measure for severe disease. In the introduction, they also suggest the previous reports of lithium inhibition of other viruses might also apply to SARs CoV2. I believe that there are several more specific and compelling reasons for expecting that lithium and other more inhibitors of GSK-3 will be beneficial in the treatment of COVID-19.

Firstly, it is important to state that a primary target of lithium involves the inhibition of GSK-3 which should inhibit SARs COV2 replication and boost the CD8 T-cell response against the virus (Taylor et al., 2016; Taylor and Rudd, 2017; Taylor et al., 2018; Rudd, 2020; Rudd et al., 2020). In the previous article (Rudd, 2020), I showed that key residues phosphorylated by GSK-3 in the nucleocapsid proteins in SARs Cov1 are conserved in SARs Cov2 (Rudd, 2020). In the case of SARs Cov1, the phosphorylation of these sites is needed for the replication of the virus. This makes it highly probable that the same sites are phosphorylated in SARs Cov2 where the inhibition SARs Cov2 replication.

Secondly, we have previously documented that GSK-3 SMIs markedly enhance CD8^+^ cytolytic T-cell (CTL) anti-viral effector functions leading to a reduction in both acute and chronic viral infections in mice (Taylor et al., 2016; Taylor and Rudd, 2017). Evidence from my lab and others also has shown that GSK-3 negatively regulates T-cell proliferation and function. This is unlike other kinases such as p56^lck^ which initiate T-cell activation (Rudd et al., 1988), GSK-3 is most active in resting T-cells where it keeps cells in a quiescent state. We have shown that the inhibition of GSK-3 by the knock-down of GSK-3 and the use of the small molecule inhibitors (SMI) markedly enhances CD8^+^ cytolytic T-cell (CTL) function (Taylor et al., 2016; Krueger and Rudd, 2017; Taylor et al., 2018). This is accomplished by down-regulating the expression of inhibitory receptors PD-1 and LAG3 and increasing the transcription of cytolytic effector molecules in CD8^+^ T-cells, granzyme B, perforin and interferon-gamma (Taylor et al., 2016; Taylor et al., 2018; Rudd et al., 2020; Taylor and Rudd, 2020).

Further, we have found that these effects preferentially affect CD8 CTLs and to a lesser extent, CD4^+^ T-cells that are more likely to contribute to the cytokine storms associated with the most severe clinical manifestations of COVID-19 (Taylor et al., 2016). GSK-3 inhibitors also induce the suppressive cytokine interleukin 10 (IL-10) in CD4^+^ T-cells which might dampen CRS in severe disease (Hill et al., 2015). Inhibition of GSK-3beta before hemorrhagic shock has also been reported to inhibit the inflammatory response and improve hepatic microcirculation and hepatocellular function (Jellestad et al., 2014). It also limits the systemic inflammatory response (SIR) in sepsis and ischaemia (Dugo et al., 2006) and has an anti-inflammatory effect during chronic inflammation that limits the severity of collagen induced arthritis (Cuzzocrea et al., 2006). Other examples of anti-inflammatory effects were also outlined in the article. Further, lithium has long been used as a treatment for bipolar and mood disorders (Jope, 2011) and recently, in Alzheimer’s disease (Matsunaga et al., 2015). As mentioned in our original commentary, the same time, lithium has certain toxic effects that can be ameliorated with proper drug dosing and monitoring (Gong et al., 2016). All risks should be understood prior to administration of any treatments and precautions taken under the close supervision of a physician. In one instance, in an area of Italy endemic with SARS-CoV2 virus that was typified by a 14% COVID-19 mortality, a one patient on lithium, in family with an instance of mortality due to COVID-19, underwent a protracted recovery (Gattner and Rybakowski, 2020).

Collectively, I believe that it is these three key arguments involving the regulation by GSK-3 that support the notion that GSK-3 inhibition by lithium or other small molecule inhibitors will concurrently inhibit SARs Cov2 viral replication, while boosting the CD8 CTL response against the virus and dampen the inflammatory cascade associated with CRSs.

## Author Contributions

The author confirms being the sole contributor of this work and has approved it for publication.

## Funding

Canadian Institutes of Health Foundation grant (159912).

## Conflict of Interest

The author declares that the research was conducted in the absence of any commercial or financial relationships that could be construed as a potential conflict of interest.
